# Patient experiences of coercion in mental healthcare: systematic review and metasummary of qualitative studies from 1991 to 2025

**DOI:** 10.1192/bjo.2026.12067

**Published:** 2026-08-06

**Authors:** Olav Nyttingnes, Tonje Lossius Husum, Solveig Helene Høymork Kjus, Tiina Överlund, Jorun Rugkasa

**Affiliations:** Health Services Research Unit, Akershus University Hospital, Nordbyhagen, Norway; Centre for Population Health, Haukeland University Hospital, Bergen, Norway; Faculty of Health Sciences and Social Care, Molde University College, Norway; Faculty of Health Sciences, Oslo Metropolitan University, Norway; Norwegian Resource Center for Community Mental Health, Norwegian University of Science and Technology, Norway; Department of Nursing Science, Faculty of Medicine, University of Turku, Finland

**Keywords:** Coercion, mental healthcare, patient experience, severe mental disorders, qualitative studies

## Abstract

**Background:**

Questions have been raised whether the patient organisations assessments of coercion – often critical – captures the breadth of patient experiences of coercion. Existing systematic reviews have not captured the full scope of reported experiences, necessitating a more comprehensive approach.

**Aims:**

To identify the existing body of qualitative studies reporting patients’ experiences of coercion; map study distribution over time, regions, and type of coercion; and synthesise broad categories of reported experiences.

**Method:**

The review protocol was preregistered (PROSPERO identifier CRD42021248744). We searched 12 databases (MEDLINE, Embase, APA PsycINFO, CINAHL, Web of Science, Sociological Abstracts, Scopus, ASSIA, Norart, SveMed+, OpenGrey.eu and Google Scholar) for qualitative studies published from 1 January 1991 to 3 June 2025. Peer-reviewed studies and approved doctoral theses were included. We used EPPI-Reviewer for screening, data extraction and coding. Study quality was assessed with a modified Critical Appraisal Skills Programme tool.

**Results:**

We included 291 studies, 18 of them from low-and middle-income countries. The most studied coercive practices were involuntary admissions, coercive measures and community treatment orders. Quality concerns included limited author reflexivity and lack of involvement of experts by experience. At least one negative experience was reported in 279 of the studies, while mixed and positive experiences appeared in 166 and 167, respectively.

**Conclusions:**

A large body of qualitative research reporting patient experiences of coercion exists, with a near-universal presence of negative experiences. Improved patient involvement in research, and more studies from low-and middle-income countries and on involuntary medication are needed.

People receiving care for severe mental health problems can sometimes be subjected to coercive practices sanctioned in mental health law. These might take the form of involuntary hospital admissions (confinement)^1^ or short-term measures to manage risk of violence, such as seclusion or restraints.^2^ Patients may also be medicated against their will, either as a type of short-term ‘chemical restraint’ for the purpose of rapid tranquilisation^3^ or symptom reduction,^4^ or as longer-term maintenance medication to prevent relapse.^5^ Further, in many jurisdictions, patients may be subjected to community coercion in the form of community treatment orders (CTOs), which are legal instruments that mandate treatment outside institutions, usually obliging patients to keep contact with services and to take maintenance medication.^6^ Coercive practices limit personal autonomy and integrity, and are reported to cause harm,^7^ with limited empirical support for benevolent effects.^8^ Marginalised groups, such as refugees and minorities, are reportedly disproportionally subjected to seclusion, restraints^9^ and CTOs,^10^ which raises concern regarding social justice.

Patient organisations and others have over the past decades advocated for reforms of mental health legislation and the reduction or abandonment of involuntary care.^11^ The abolitionist stance of leading patient organisations were incorporated into the United Nations Convention on the Rights of Persons with Disabilities (CRPD), and the convention severely limits the scope of coercive practices in signatory countries.^12^ Others, including many professionals, warn that the critical views of these organisations are not representative of, nor in the best interest of, the wider population with severe mental health problems.^13^ As a result, some have argued that the CRPD compromise the well-being of people with mental health problems, who might deteriorate in the absence of involuntary care.^14^ In the tradition of ‘benign paternalism’, it has been argued that coercion should be used with great care, and that involuntary admissions should only be used in situations and ways in which the afflicted person might come to agree with or be grateful for in retrospect – the so called ‘thank you theory’.^15^

Disagreements about the appropriate balance between of professionals’ concerns and patient autonomy are therefore partly about patients’ experiences with and appraisals of coercive care. The view that those affected by coercion are more accepting of it than the impression conveyed by critical patient organisations also merits consideration.

## Reviews of patients’ experiences of coercion

It is sometimes argued that there is insufficient research on the perspectives of people subjected to coercive mental healthcare. However, a range of studies have been published since the early 1990s. In 2012, a review of quantitative studies included 18 studies with a total of 3489 patients, and found that 74% of patients under involuntary care experienced their treatment as coercive.^16^ Quantitative studies have mainly assessed individual views on predefined items, which means perspectives grounded in respondent’s own experiential backgrounds may not be captured. Such perspectives, which seem essential to gain a deep understanding of patients’ appraisal of coercive care, is best explored by qualitative methods. An accumulating number of qualitative studies have actively sought experiences of coercive care expressed freely and situated in individual contexts, and patients also report such experiences when asked more generally about admissions or treatment. Such qualitative studies have enabled the accumulation of experience-near insights, and systematic literature reviews report a range of experiences, dominated by negative ones.^17,18^

When examining which studies were included in existing systematic reviews of qualitative studies of experiences of coercion, we observed a lack of convergence. For instance, a review by Akther et al,^18^ which covered both involuntary admissions and seclusion and restraints, only included seven of the 26 studies of seclusion and restraints in Tingleff et al’s review^19^ that was published 2 years previously. Pridham et al^20^ included 11 qualitative studies of patient experiences of CTOs, whereas Corring et al’s review^21^ on the same topic included 22 data collections (reported in 28 studies), and only seven studies were included in both. Such discrepancies might be partially explained by different search strategies: most reviews search PubMed or MEDLINE, Embase and PsycINFO, but the use of other databases such as CINAHL and Web of Science varies. Consequently, it seems that existing systematic reviews have not been comprehensive enough to encompass all relevant studies covering the topic of interests, which means they fail to cover the breadth of qualitative reports of patients’ experience. This is at odds with methodological recommendations to include all studies with relevant data in qualitative reviews, regardless of methodological or reporting limitations.^22^

## Aims

Given these limitations, there is a need for a more all-encompassing systematic review of published qualitative studies, to assess the academic literature of patients’ experiences of coercive practices more fully. In this article, we present, as a first step toward this agenda, a metasummary of the literature, based on a broad search that yielded a wide and varied body of studies. Metasummaries are designed to provide an overview of a field of research, highlight underresearched topics, inform research priorities and summarise the main patterns of manifest findings.^22^ The metasummary presented here has the following aims:

- to identify the existing body of qualitative studies on patient experience of coercion;
- to give an overview of when and from which countries studies are published, the types of coercive practices being studied, data collection methods, analytic approaches and quality;
- to provide a descriptive overview by extracting from each study whether negative, positive and/or mixed experiences were reported by participants.

## Method

### Search strategy

The review was registered in the International Prospective Register of Systematic Reviews (PROSPERO) under identifier CRD42021248744, on 14 May 2021. We cooperated with the medical library at Akershus University Hospital in combining and modifying search terms and strategies from earlier reviews.^18–21,23^ We combined search blocks for ‘topic’ (coercion), ‘treatment field’ (mental healthcare) and ‘research methods’ (qualitative). We performed test searches, mainly in MEDLINE, PsycINFO and CINAHL, and cross-referenced the search results with studies included in the previous reviews.^18–21,23^ It proved difficult to find terms for the ‘topic’ search block that covered all studies included in these reviews. Similarly, the ‘method’ block required a comprehensive set of terms to account for the diverse terminology used to describe qualitative approaches. We therefore iteratively broadened our search, thus prioritising recall of many studies over search precision, to reduce the risk that eligible studies were not captured. The medical library adapted the search blocks in MEDLINE to Embase, APA PsycINFO, CINAHL, Web of Science, Sociological Abstracts, ASSIA, Norart, SveMed+, OpenGrey.eu, Scopus and Google Scholar. Thesauruses and optimal search techniques vary between databases. The search combination of keywords, MeSH terms and free-text search terms was therefore adapted to each database. The original searches were completed on 2 November 2021, with an update search on 3 June 2025. Metadata extraction took place during full-text screening, and results were coded after inclusion. After the final inclusion decisions, we conducted a DOI-based citation search of the references in the included studies, using Citation Chaser.^24^ The complete search strategies for all databases are available in the Supplementary Material available at https://doi.org/10.1192/bjo.2026.12067.

### Inclusion and exclusion criteria

We included peer-reviewed articles and postdoctoral theses, published between 1 January 1991 and 3 June 2025, that used qualitative methods and reported patients’ experiences of coercion. We included studies in English or Scandinavian languages, reflecting our language skills. To reduce the risk of self-selection, we excluded studies without a sampling strategy, such as case studies and autoethnographies. We included studies that did not have patient experiences of coercion as an explicit aim or focus, but that reported such experience from, for instance, patient experiences of in-patient care or of living with a severe mental disorder, but limited inclusion to coercive practices sanctioned in law. Where more than one study reported from the same data collection, all were included, but metadata was recorded only once, which is why we report a smaller number of data collections than studies.

### Screening and inclusion procedures

The librarian collated all search results in Endnote and removed duplicates. We then imported the resulting records into the EPPI-Reviewer 6 tool designed for systematic reviews (EPPI Centre, UCL Social Research Institute, University College London, UK; https://eppi.ioe.ac.uk/cms/er4/About/About-EPPI-Reviewer) and added terms that assisted inclusion and exclusion to the EPPI interface. In the original search, O.N., T.L.H. and J.R. double-screened 500 records on title and abstract and met twice to discuss discrepancies, reach consensus and calibrate the approach after each round. For the updated search, T.Ö. and O.N. double-screened 100 papers and similarly met and calibrated the approach. Full-text PDFs for included studies were uploaded to EPPI-Reviewer. Some studies had to be digitalised, and four were obtained by contacting the authors. The screening of full texts commenced with double-screening of approximately 10% of studies against the inclusion criteria in both the original and updated search, after which all authors met to resolve disagreements and calibrate the final inclusion threshold. The citation search and screening was done by O.N. Although software supported the workflow, all screening, inclusion and text extraction was done by the authors without the use of automated text-mining or artificial intelligence.

Box 1 Coercive practices in mental healthcare.

**Table:** long description.

**Involuntary admission** is admission to hospital that is sanctioned in law, either against the will of the patient or for those lacking decision-making capacity who represent risk to themselves or others.^25^ **Forensic care** takes place in the interface between mental healthcare and penal code, usually initially decided by the penal system following a serious legal offence by the patient, often in the form of long-term detention in secure in-patient settings.^2^ **Coercive measures** are measures used to control or reduce the risk for a patient’s aggressive acts or self-harm. Seclusion separates the patient from other patients and/or staff, to various degrees. Restraints restrict movement with either mechanical restraints (such as belts, jackets or limb cuffs), physical restraints (manually holding the patient) or chemical restraints (psychotropic drugs or rapid tranquilisation).^25^ **Community treatment order** is a legal order that mandates a patient to adhere to treatment in the community.^25^ **Involuntary treatment** is forced treatment of a patient, usually involving medication aimed at symptom reduction or relapse prevention, but can also include other treatments (e.g. electroconvulsive therapy or nasogastric tube feeding).^25^ **Tribunal/review board procedures** are formal hearings by an authority or judicial body, the purpose of which is safeguarding that clinical decisions conform to legal requirements, often following patient appeal or routine re-examination.^26^

### Extraction of metadata

Metadata were extracted for each data collection. This included year of publication, country and methods for data collection (interview, focus group, observation, open-ended questionnaire items, other/mixed) and analysis (content analysis, grounded theory, phenomenological (including phenomenographic), narrative, other/unspecified), and time of data collection relative to the experience (during the coercive event, within 30 days, retrospectively, mixed/unclear). Type of coercion categorised studies into types commonly used in the literature and was coded according to the descriptions in Box 1. It is worth noting that these practices are not necessarily mutually exclusive or independent. For example, involuntary admissions are often prerequisites for both coercion measures and later CTOs. CTOs are also often used in combination with compulsory maintenance treatment, and tribunal hearings may concern any coercive practice. Studies that aimed to report experiences of more than one type of coercion were coded as ‘mixed type’.

We extracted the sample size for each data collection. Where there were mixed samples (e.g. patients with and without experience of coercion, or studies also including health professionals or family carers), we only counted the number of patients that reported experiences from coercive events. If the sample size was not reported or the number of relevant patients could not be deduced, they were not included in the sample size counts. For studies of forensic patients or high-security services, all patients were regarded as reporting from care under coercion.

To map the socio-geographical spread of studies, we assigned each data collection to a world region and an associated gross national income level per capita, based on its country of origin, following The World Bank’s classification of gross national income per capita for 2019.^27^

### Quality assessment

We assessed study quality with an amended set of the ten Critical Appraisal Skills Programme (CASP) items,^28^ and following Long et al,^29^ we added an alternative of ‘partly’ to the original scoring of ‘yes’/‘no’/‘can’t tell’, to increase nuance.

For CASP items 2 (is qualitative methodology appropriate), 3 (appropriate research design), 4 (appropriate recruitment strategy), 5 (data collection addressing the research issue) and 9 (clear statement of findings), a score of no would violate our inclusion criteria, so these items were evaluated as acceptable (yes or partly) by being included. We did not evaluate ethical issues (CASP item 7).

The remaining quality items applied were scored as follows: CASP item 1 (clarity of aim) was scored either as a ‘stated aim of investigating experiences of coercion’, ‘unclear aim’ or ‘other aim’. CASP item 6 (adequate consideration of relationship between researcher and participants) was scored with ‘yes’ if the discussion included reflections on researcher bias and/or potential influence on participants, ‘partly’ if reflection was weak or insufficient, and ‘no’ if absent. CASP item 8 (analytic rigorousness) was scored as high, intermediate or low. Finally, we rated overall value (CASP item 9) for the aims of our review. Here, we considered the density of relevant findings, and how findings and analyses contributed to the pool of knowledge of patient experience of coercion in mental healthcare. For example, a study contributing relevant findings, but with limited analytic depth or unspecified analytical approach, was scored with ‘intermediate value’. Finally, we added an item of involvement of experts by experience^30^ (experts by experience in research team, reference group with lived experience, no/not reported) to the included CASP items. Our reasoning was that power disparities in research processes may augment risks of othering or epistemic injustice, which might be reduced or overcome by including experts by experience. Our quality items were not double-scored, therefore reliability cannot be reported.

### Extraction and coding of findings

Following established methodology for synthesising qualitative research,^21^ by ‘finding’ we here mean what appears in studies’ results section (often as citations or paraphrasing of participants words), and also the relevant authors’ interpretations of their overall results (often as discursive statements in the discussion section). We constructed a coding interface for full texts in EPPI-Reviewer, which allowed for line-by-line coding of patient experience reported in the result and discussion sections. Findings could refer to individuals or groups of patients. In studies that reported findings from both voluntary and coerced patients, we only coded findings attributable to experiences of care under coercion. Also, in studies that included staff and family carers in addition to patients, we only coded findings attributable to patients; findings with absent or unclear attributions were not coded. From this, we classified each study as whether one or more patients reported negative, positive and/or mixed experiences of coercion, to provide a descriptive overview of findings across the body of literature. For examples of how these were classified, see Box 2. The ‘mixed’ code was applied to findings that either indicated an ambivalent description or combined positive and negative evaluations of a practice or situation. Codes for more in-depth exploration will be reported in future work.

Box 2 Examples of findings sorted as three types of experience (all entries are shown verbatim from the cited studies).

**Table:** long description.

Negative patient experience	… others felt strongly that being on a CTO was a negative experience and not about care, but rather about being monitored or under surveillance by the team, which may implicitly feel disempowering: ‘[The CTO] sort of demoralizes you in some aspects because it takes away your choices, your decision-making in some respects. [pause] Its compulsory medication which is not always the right thing I don’t believe.’^31^
	Patients explained that they experienced seclusion as being in a prison because in their opinion only prison inmates are locked up and are deprived of freedom of movement. The patients who were imprisoned prior to their experience of being secluded clearly revisited their imprisonment when they were secluded. The memories and negative emotions related to imprisonment were triggered by the similarity for them between a prison cell and a seclusion room.^32^
Mixed experience	Overall, the sense of lacking choice and control, the emphasis placed on medication and the threat of hospitalisation were more dominant concerns in the consumer interviews but, on balance, having supports was also an important element of being on a CTO for some consumer participants.^33^
	Being committed against one’s will was sometimes experienced as terrible, while at the same time secure and necessary.^34^
Positive experience	Patients greatly appreciate continuous observation as it seems to achieve the right balance of feeling cared for while still remaining autonomous. ‘I’ve had it a few times that a nurse offered me to talk a little or that I could reach out to them for a chat and I liked that very much. In general, that there are people around you and don’t harass you and don’t want anything but just have a look at what you’re doing, how you are. […] I’ve always found that pleasant.’^35^
	Although, under s117 of the Mental Health Act, support should be provided according to need, service users tended to equate the CTO with receiving higher levels of support and hence were often keen to stay on it. This was the case for one young man, for example, who described the CTO as a guarantee of support and monitoring (which he felt had been lacking in the past, leading to isolation and deterioration in his mental health): ‘I’m on my own. I need someone like a social worker to come and drop off my medication, a doctor to come in every six months to see me, to make sure I’m alright. I’ve got no-one. People like me need things like that.’^36^

CTO, community treatment order.

### Software

We developed a spreadsheet for extraction of metadata in Microsoft Excel 365 2402 for Windows, analysed them in R version 4.1.2 for Windows (R Foundation for Statistical Computing, Vienna, Austria; https://www.R-project.org) and used GGplot2 version 4.0.1 for graphs (for Windows; Hadley Wickham et al., https://ggplot2.tidyverse.org/). In cases where more than one study was published from the same data collection, metadata are only counted once in the summaries reported below, unless noted otherwise. As reported above, EPPI-Reviewer 6 was used for screening and text extraction.

Below, we present tables and figures from the metasummary, including numbers of studies with positive, negative or mixed views. By this, we mean that one or more patient in that study reported, for instance, a mixed experience that the author included in the article and we extracted as a finding. Although the share of studies reporting positive, mixed and negative experience may shed some light on the pervasiveness of such experiences as a whole and in certain circumstances, such results should not be read as indicating a quantified direction or weight of views among participants in a study or group of studies, and the absence of a type of experience could both indicate that they were not reported by patients or that such findings were not disseminated by the authors. The qualitative approaches across the included studies, and our coding approach, precludes any meaningful such ‘counting’ of types of experience or patients with certain views.

### Reflexivity

The review formed part of the Reducing Coercion in Norway (ReCoN) project, funded by the Research Council of Norway, which sought to increase our understanding of how the political aims to reduce coercion in mental healthcare may be realised. The motivation for producing the present metasummary – beyond the stated research questions – was to pave the way for detailed analyses of the findings of the included studies, to contribute to identifying approaches to prevent, reduce or ameliorate coercive experience in mental healthcare. The author team includes an expert by experience and physicist (S.H.H.K.), a sociologist (J.R.), two clinical psychologists (T.L.H. and O.N.) and a psychiatric nurse (T.Ö.). The authors have published extensively on coercion in mental healthcare, including patients’ perspectives, and are well versed in qualitative methodology. Our different professional training, experience and competencies meant a range of perspectives informed and enriched our discussions on coding, data extraction and interpretation.

## Results

**Fig. 1:**
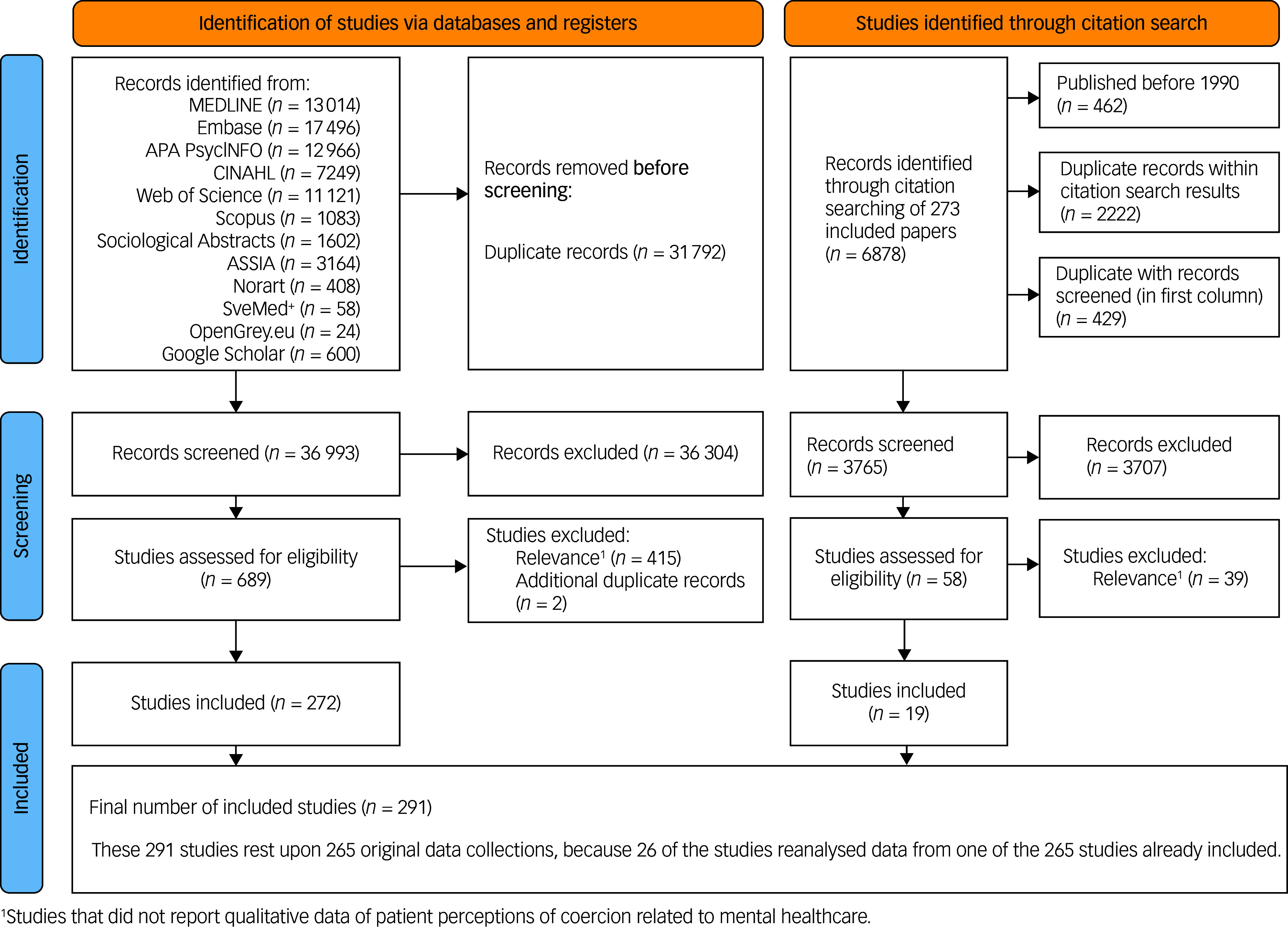
Fig. 1 long description. Preferred Reporting Items for Systematic Reviews and Meta-Analyses flow diagram of search, screening and inclusion.

The search results are shown in the Preferred Reporting Items for Systematic Reviews and Meta-Analyses diagram in Fig. 1. After removing duplicates, 36 993 records remained for screening on title and abstract. The first double-screening of 513 records resulted in 17 disagreements (3.3%), and once these were resolved, the second double-screening of a further 510 records resulted in ten disagreements (2.0%) and therefore we considered the threshold calibrated. Once the remaining records were screened, the authors met to discuss and decide on inclusion of 170 records marked for joint consideration.

For 689 records included for full-text screening, we acquired PDFs or paper copies. We double-screened the first 57 of the full texts and met to resolve 12 disagreements (21%). We then established a liberal inclusion policy to ensure relevant findings were not missed. Having screened all full texts, the authors convened to resolve 32 studies marked for joint consideration, and this resulted in 272 included studies reporting from 247 data collections. The references of all studies were then assessed with Citation Chaser,^24^ which identified an additional 3765 records after duplicate removal. The assessment of these resulted in the inclusion of an additional 19 studies, only one of which used data from an already included data collection. The final data-set therefore comprised 291 studies, reporting from 265 data collections. Studies in English dominated, with only five studies in Scandinavian languages. Full references and metadata for all 291 studies are available as Supplementary Material.

### Time trend and geographic distribution

**Fig. 2:**
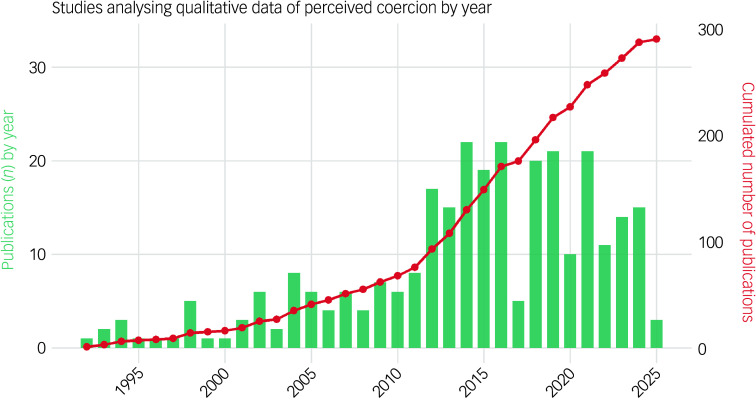
Studies analysing qualitative data of patient experience of coercion by publication year. Left *y*-axis: number of studies published each year, indicated by the bars. Right *y*-axis: accumulated number of studies over time, indicated by the continuous line.

Figure 2 depicts trends in the 291 studies over time. The red line shows the cumulative number and the green bars show annual numbers from 1990 onward. There was a marked increase in studies from 2012, with 74% of all included studies published between 2012 and June 2025.

We were able to identify the number of included patients reporting their experience of coercion in 200 of the 265 data collections, which combined included 3853 patients. In five data collections, patient participant numbers were not reported, and for 60 data collections (with 2784 patient participants), we could not find or deduce the number of patients with experience of coercion; consequently, participant numbers from these studies are not reported in the tables or text. Fifty-three of these 65 data collections were not designed to investigate experiences of coercion.

The geographic distribution of the included data collections across countries and regions is presented in Table 1, along with combined sample size per country and the classification of income level of each country. Although our inclusion criteria opened for data collections from all world regions, 61% were from Europe and almost a third were from the UK alone. The share of data collections and patient participants under coercion from low-and middle-income countries were 6.8 and 5.7%, respectively.

**Table 1:** Data collections and participants by country and country income level classification^a^ Table 1 long description.

Region	Country^a^	Income level^b^	Data collections, *n* (%)	Participants,^c^ *n* (%)
Africa	Ethiopia^d^	Low	1 (0.4)	–
	Nigeria	Lower middle	1 (0.4)	17 (0.4)
	South Africa	Upper middle	5 (1.9)	74 (1.9)
Asia	China	Upper middle	3 (1.1)	47 (1.2)
	Hong Kong	High	1 (0.4)	30 (0.8)
	India^d^	Lower middle	1 (0.4)	–
	Indonesia	Upper middle	2 (0.8)	48 (1.2)
	Iran	Upper middle	3 (1.1)	29 (0.8)
	Israel	High	1 (0.4)	15 (0.4)
	Japan	High	1 (0.4)	18 (0.5)
	Pakistan^d^	Lower middle	1 (0.4)	–
Europe	Austria	High	1 (0.4)	15 (0.4)
	Belgium	High	2 (0.8)	29 (0.8)
	Denmark	High	7 (2.6)	53 (1.4)
	Finland	High	5 (1.9)	72 (1.9)
	Germany	High	3 (1.1)	41 (1.1)
	Greece	High	1 (0.4)	14 (0.4)
	Ireland	High	7 (2.6)	165 (4.3)
	Multiple^d^	High	1 (0.4)	–
	Netherlands	High	7 (2.6)	56 (1.5)
	Norway	High	23 (8.7)	217 (5.6)
	Spain	High	3 (1.1)	19 (0.5)
	Sweden	High	15 (5.7)	114 (3)
	Switzerland	High	3 (1.1)	83 (2.2)
	UK	High	84 (31.7)	1573 (40.8)
Oceania	Australia	High	23 (8.7)	245 (6.4)
	New Zealand	High	3 (1.1)	52 (1.3)
Latin America	Brazil	Upper middle	1 (0.4)	3 (0.1)
USA/Canada	Canada	High	23 (8.7)	352 (9.1)
	USA	High	33 (12.5)	472 (12.3)
			265 (100)	3853 (100)

a. The results reflect studies published in English or in Scandinavian languages.

b. The World Bank Analytical classifications of gross national income per capita in 2019 (Atlas methodology).

c. Patients with experience of coercion reported in 200 data collections. For five data collections, the number of participants was not reported, and for 60 data collections, the number of patients with experience of coercion could not be deduced and is therefore not included in the participant numbers reported here.

d. We were unable to deduce the number of patients under coercion in the data collection from this country.

### Type of coercion and data collection methods

Metadata on the types of coercive events, data collection methods and analytic approaches are shown in Table 2. The most frequent type of coercion, with 73 data collections, was involuntary admissions, followed by 50 data collections on coercive measures, 39 on CTOs and 16 on forensic care. Fourteen studies focused on compulsory treatment to reduce symptoms, including electroconvulsive therapy, tube feeding and medication. Only two studies had experience of involuntary maintenance medication as a study aim. Finally, 66 data collections had multiple foci or could not be categorised into these specified types of coercion.

**Table 2:** Characteristics of 265 included data collections Table 2 long description.

Characteristic	Data collections, *n* (%)	Participants, *n* (%)^a^
Type of coercion reported from		
Involuntary admission	73 (27.5)	1008 (26.2)
Seclusion and restraints^b^	50 (18.9)	757 (19.6)
Community treatment order	39 (14.7)	641 (16.6)
Forensic care	16 (6)	314 (8.1)
Compulsory treatment	14 (5.3)	85 (2.2)
Tribunal/review board procedures	7 (2.6)	110 (2.9)
Mixed or other^c^	66 (24.9)	938 (24.3)
Time of data collection relative to the coercive event		
While under coercion	63 (40)	1090 (28.3)
Within 30 days of the event	27 (10.2)	322 (8.4)
Retrospectively	106 (40)	1603 (41.6)
Mixed or unclear	69 (26)	838 (21.7)
Data collection method		
Individual interviews	196 (74)	2519 (65.4)
Focus group interviews	28 (10.6)	479 (12.4)
Ethnographic observation	6 (2.3)	41 (1.1)
Open-ended questionnaire items	5 (1.9)	251 (6.5)
Mixed or other^d^	30 (11.3)	563 (14.6)
Analytic approach^d^		
Thematic	120 (41.2)	1976 (51.3)
Phenomenological	42 (14.4)	342 (8.9)
Grounded theory	36 (12.4)	515 (13.4)
Content	30 (10.3)	417 (10.8)
Narrative	12 (4.1)	44 (1.1)
Unspecified or other^e^	51 (17.5)	559 (14.5)
Patient or more groups sampled in the data collection		
Only patients	181 (68.3)	2608 (67.7)
Patients, carers and/or staff	84 (31.7)	1245 (32.3)

a. The participant number reflects 200 out of 265 included data collections because five data collections did not report the number of participants, and in 60 studies the number of patients under coercion could not be deduced.

b. Seclusion (segregation from other patients with or without presence of staff); physical, mechanical or chemical restraints (see also coercive measures in Box 1).

c. Sixty-six data collections reported data from a combination of types of coercion, including involuntary admission (*n* = 57), compulsory treatment (*n* = 45), seclusion and restraints (*n* = 26) or community treatment orders (*n* = 7). This commonly followed an aim where experiences with several coercion forms were relevant (e.g. retrospective report of experience of coercion, a focus on advance care planning or the role of carers in recovery). Three data collections focused on police assistance or transportations, and one study each on supported housing and assessment for the mental health act.

d. Includes all 291 studies and not data collections.

e. Twenty-four studies did not specify analytic approach, and 27 studies were spread across various methods such as critical ethnographic, general inductive, etc.

Individual semi-structured interviews were the most common data collection method, employed in 196 data collections, followed by focus groups in 28. Data collections using ethnographic observation were rare, with only six instances. The most common analytic approach was thematic analysis, with 120 studies; phenomenological, grounded theory or content analysis were each used with 42, 36 and 30 studies, respectively. Fifty-one studies lacked information that enabled allocation to an analysis category, and 26 of these were interview studies. A total of 149 studies reported the number or share of participant gender for patients under coercion, and in these studies, 42% of participants were women.

Data collection conducted while the patient was subjected to coercion was rare for coercive measures (one study), but more frequent for involuntary admissions (20 data collections, 27%), and this was common in studies of CTOs (19 data collections, 49%) and forensic care (11 data collections, 69%).

### Quality of included studies

The assessment of study quality is summarised in Table 3 and shows several quality concerns. A total of 150 (52%) studies lacked author reflexivity, 209 (79%) lacked involvement of experts by experience and 63 (22%) were rated as low analytic quality. Studies that combined reports from patients and other groups were more frequently rated as low analytic quality (19% compared with 33% for studies with only patient participants). Studies with unspecified analytic method tended to be older, with a median publication year of 2007.5 compared with 2016 for the remaining studies; publication year difference was less for studies that included experts by experience (median publication year of 2018 compared with 2015 for studies without experts by experience), and for studies that included some degree of author reflexivity (median publication year of 2017 compared with 2014 for studies that did not include author reflexivity).

**Table 3:** Quality assessment of the 291 included studies Table 3 long description.

Quality appraisal item	Studies, *n* (%)	Participants, *n* (%)^a^
Consideration of relationship between researcher and participant		
Yes	67 (23)	778 (20.2)
Partly	74 (25.4)	1034 (26.8)
No	150 (51.5)	2041 (53)
Analytic rigorousness		
High	46 (15.8)	545 (14.1)
Intermediate	182 (62.5)	2310 (60)
Low	63 (21.6)	998 (25.9)
Involvement of experts by experience^b^		
Present in the research team	41 (15.5)	886 (23)
Consulted in a reference group	15 (5.7)	147 (3.8)
No/not reported	209 (78.9)	2820 (73.2)
Clear aim to investigate experiences of coercion^c^		
Clear	167 (63)	2866 (74.4)
Unclear	7 (2.6)	75 (1.9)
Other aim than experience of coercion	91 (34.3)	912 (23.7)
Overall value for this review		
High	63 (21.6)	957 (24.8)
Intermediate	132 (45.4)	1771 (46)
Low	96 (33)	1125 (29.2)

a. The participant number reflects 200 out of 265 included data collections (five data collections did not report the number of participants, and in 60 studies, the number of patients under coercion could not be deduced).

b. Involvement of expert by experience refers to 265 data collections.

c. Study aim refers to 265 data collections.

### Reported experiences of coercion

All 291 included studies reported on patients’ experiences of coercive practices. Of these, 286 studies included patient appraisal of a coercive practice or experience. The remaining five studies compared coercive practices or reported suggested alternatives that did not fit into our categories.

**Fig. 3:**
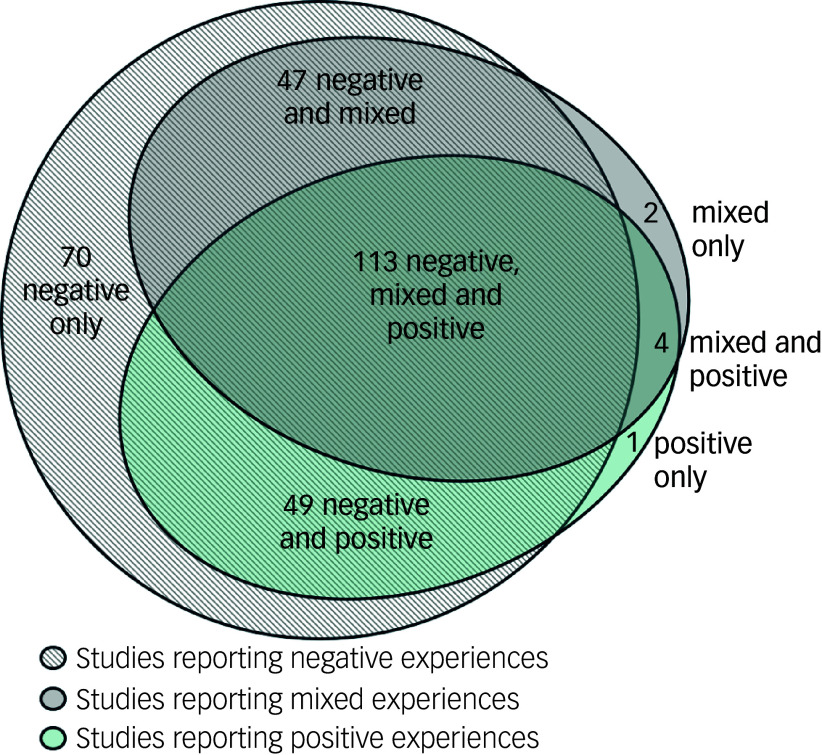
Venn diagram illustrating studies reporting at least one positive, mixed or negative experience, and their overlap.

Three-quarters of the studies (213 studies) were coded as having more than one of these types of patient experience, whereas 73 studies only reported one type of experience. The Venn diagram in Fig. 3 illustrates these types of experiences as coded from each study, and their overlap. At least one negative experience (striped area) was reported in 279 studies, whereas seven studies did not report any negative experience; 166 studies reported at least one mixed experience and 167 reported at least one positive experience.

Among the six countries with ten or more included data collections, we found that positive experiences were reported in around half of the included papers from Australia, Canada, Norway, Sweden and the UK, whereas in three-quarters of studies from the USA, one or more patients reported positive experiences.

We studied the reporting of positive, mixed and negative experiences of a coercive event as related to the timing of data collection for studies of involuntary admission. This is because a CTO is often considered a milder form of coercion, and such studies could skew the results toward positive experiences during coercion, whereas for forensic services, the opposite might be the case. The results in Table 4 show that negative experiences dominate involuntary admissions, and their share remains the same regardless of timing, whereas 2.5% more retrospective studies reported positive experiences compared with data collected during involuntary admission.

**Table 4:** Patient experiences of involuntary admission, either during or retrospectively Table 4 long description.

Time of data collection relative to involuntary admission (*n* = 73)	Data collections reporting the experience, *n* (%)		
	Positive	Mixed	Negative
During involuntary admission (*n* = 20)	12 (60)	11 (55)	19 (95)
After the involuntary admission ended (*n* = 40)	25 (62.5)	27 (67.5)	38 (95)
Mixed (*n* = 13)	4 (30.8)	4 (30.8)	11 (84.6)

## Discussion

We identified 291 studies published from January 1991 to 3 June 2025 that used appropriate methodologies for investigating personal experiences and reported patients’ experiences of coercion in mental healthcare. Almost three-quarters of these were published between 2012 and 2025, indicating a soaring interest from the research community toward patients’ own considerations of coercive practices. This could reflect the expanding focus on patients’ rights to autonomy and self-determination, signalled by, for example, the CRPD^37^ and the World Health Organization’s QualityRights initiative.^38^

The regional distribution of studies is of concern, with only 21 studies from Africa, Latin America and Asia combined. Of these, five were from South Africa, and three each from China and Iran. The vast majority of studies were from high-income countries in Europe, Oceania and North America. This suggests that patients’ evaluation of coercion is higher up on the research agenda in high-income Western countries, with extensive, and often public, services governed by mental health legislation.

Only two data collections specifically investigated experiences of involuntary maintenance medication, although experiences of such medication were also reported in studies primarily focusing on involuntary admissions, CTOs and forensic care. This low number might be unexpected as such treatment is relatively common, applied in both in-patient and out-patient services (in particular for patients on CTOs) and might last for years.^39^ This practice is also heavily criticised by patient organisations and their advocates.^11^ Tailored sampling strategies and in-depth exploration may improve understanding of this practice.

### Study quality and the relative absence of expertise by experience

There were some quality concerns across this large body of studies. Half of the studies lacked reflexivity, including adequate consideration of interactions between researchers and participants, such as power disparities, stigma and interviewer influence. Such reflexivity is a hallmark of the quality of qualitative research,^30^ and is particularly important here because coercion by definition involves power disparities and might be accompanied by experiences of powerlessness or expressions of opposition.^40^ A total of 79% of studies did not report any involvement of experts by experience. This is of concern, given longstanding complaints about epistemic injustice^41^ and a history of patients’ experiences of being portrayed as passive subjects, problems or victims.^42^

However, study quality seems to have improved over time, and the large total number of studies may partially offset these limitations of individual studies.

### The importance of uniform key terms

The number of included studies confirmed our concerns that previous reviews might have missed relevant studies, and that a broad search strategy is necessary for an exhaustive representation of qualitative studies on mental health coercion. As we refined our search, we increasingly widened the net to catch studies identified by existing reviews. In particular, single-topic or method terms did not perform well. Nevertheless, ‘coercion’ and ‘qualitative’ were used in many studies, and we suggest that the research community adopt these terms in titles, abstract or keywords – even when other terms might be preferable for theoretical reasons – to facilitate search and retrieval in future reviews. To facilitate such reviews, our search terms and database-specific adaptations, as well as a full list of the included studies, can be found in Supplementary Material.

### Negative experiences of coercion are ubiquitous

Although 40% of studies reported both positive, negative and mixed experiences, 98% reported at least one negative experience. This applied across countries, types of coercive events, modes of study recruitment, data collection methods, timing of data collection relative to coercion, analytical approaches and regardless of study quality. It also came up in studies that were not specifically investigating coercion. Moreover, it concurs with the conclusions in more selective reviews.^18,19,22,43^ This suggests that it is likely that future qualitative studies will continue to report negative patient evaluations of coercion in mental healthcare.

Our strategy led to the inclusion of 91 studies (34%) that did not set out to study experiences of coercion. Some of these included only a small number of relevant findings. However, including them demonstrates that patients who are invited to discuss experiences of mental healthcare raise concerns over coercive practices even when this is not the aim of the study, which underlines a persistent and emotive impact of coercion on those who have experienced it. Despite fewer relevant findings and less analytic depth regarding coercion in these studies, their content was, on the whole, consistent with the rest of our material.

The recorded patient experiences for each study regarding involuntary admissions showed only minimal differences between retrospective studies and those conducted during the event. If marked differences had been observed, this could have offered a tentative and indirect support for the ‘thank you theory’; that coercion commonly receives retrospective acceptance despite prospective opposition.^15^ Nevertheless, more retrospective studies reported mixed patient experiences, and might indicate increasingly nuanced patient evaluations over time.

Many studies also reported positive experiences of coercive care, which is in line with previous reviews.^18,22^ This indicates that not all patients think all coercive practices should be abandoned. Future in-depth analyses of patient experiences might shed light on what they consider better, or alternative, practices in situations where coercion is currently used, and may inform development of new care practices and treatment ideals. This should be a topic for future studies.

### Strengths and limitations

The main strength of this review is the broad search that identified a high number of relevant studies. There is nevertheless a risk that some studies were omitted. Studies not in English or Scandinavian languages were excluded, which may have skewed the geographic profile of included studies, in particular from low-and middle-income settings. We screened on title, abstract and keywords, which means studies where relevant terms only figured in the results would not have been captured. We double-screened a proportion of abstracts and articles, with high reliability. Despite our efforts to ensure interrater reliability and extensive discussions of metadata and coding, the use of single-screening for most papers, as well as for study quality assessment and line-by-line extraction of findings, may have increased the risk of bias. We cannot rule out some omissions, mistakes and punching errors, but such mistakes were not found during later discussions of text extractions or other results.

Although the author team had varied and relevant expertise, not having a trained psychiatrist in the research team might have affected our approach. As for all qualitative metasummaries, the extracted findings have been filtered through several stages of research processes, including potential bias in funding, researcher interests, data collection, interpretation and dissemination.

Exploring differences based on type of coercion or between demographic or diagnostic subgroups was beyond the scope of the article, as were more in-depth analyses of the diverse experiences reported.

This metasummary shows that there is not a lack of knowledge of how patients experience coercion in mental health services, at least from high-income countries. The consistency across a broad range of studies, combined with the sheer volume of included studies, leads us to conclude that negative experiences represent a salient, frequent and enduring appraisal by those subjected to coercion. As such, the critical stance of patient organisations toward coercion seems to be rooted in experiences that are ubiquitous. This gives reasons to continue to critically assess current justifications for coercion, and for all stakeholder parties and policy makers to continue to develop and implement practices that patients wish to receive voluntarily.

More research is needed from low-and middle-income countries. We think future studies also should examine experiences of tribunal procedures, involuntary maintenance medication and more specific aspects of coercion, such as precursors, initiation and revocation, as well as whether coercion is appraised differently by subgroups. More in-depth exploration of the existing literature should focus on what patients see as potential substitutions for coercive practices, and factors that might mitigate how coercion is experienced.

## Supporting information

10.1192/bjo.2026.12067.sm001 Nyttingnes et al. supplementary material 1 Nyttingnes et al. supplementary material

10.1192/bjo.2026.12067.sm002 Nyttingnes et al. supplementary material 2 Nyttingnes et al. supplementary material

## Data Availability

The search terms and overview of included studies and metadata are available in editable form as supplementary material. Further details can be provided upon reasonable requests to the corresponding author.

## Supplementary material

The supplementary material is available online at https://doi.org/10.1192/bjo.2026.12067

## Fig. 1 Long description

The flowchart is divided into two main sections: Identification of studies via databases and registers, and Studies identified through citation search. The Identification of studies via databases and registers section lists records identified from various databases such as MEDLINE, Embase, APA PsycINFO, CINAHL, Web of Science, Scopus, Sociological Abstracts, ASSIA, Norart, SveMed+, OpenGrey.eu, and Google Scholar. Duplicate records are removed before screening. The Screening section shows records screened, records excluded, studies assessed for eligibility, and studies included. The Studies identified through citation search section includes records identified through citation searching, removal of duplicate records, records screened, records excluded, studies assessed for eligibility, and studies included. The final number of included studies is 291, which rest upon 265 original data collections.

Navigate back to Fig. 1.

## Table 1 Long description

The table presents data collections and participants by country and income level classification across various regions. It includes four columns: Region, Country, Income level, Data collections, n (%), and Participants, n (%). The regions listed are Africa, Asia, Europe, Oceania, and Latin America, with the USA/Canada grouped together. Each row provides specific data for each country, including the number of data collections and participants, along with the income level classification. Notable entries include high participant numbers in the UK, USA, and Canada, and a significant concentration of data collections in Europe.

Navigate back to Table 1.

## Table 2 Long description

The table presents characteristics of 265 data collections on coercive events, organized into several categories. It has 15 rows and 3 columns. The columns are labeled ‘Type of coercion reported from’, ‘Data collections, n (%)’, and ‘Participants, n (%)’. The rows are grouped into categories such as ‘Type of coercion reported from’, ‘Time of data collection relative to the coercive event’, ‘Data collection method’, ‘Analytic approach’, and ‘Patient or more groups sampled in the data collection’. Each row lists specific types or methods along with corresponding data collections and participant counts. For example, under ‘Type of coercion reported from’, involuntary admission has 73 data collections (27.5 percent) and 1008 participants (26.2 percent). The table provides detailed counts and percentages for each category, offering a comprehensive overview of the data collections’ characteristics.

Navigate back to Table 2.

## Table 3 Long description

The table summarizes the assessment of study quality across various criteria, detailing the number of studies and participants involved. It has 6 rows and 3 columns. The columns are labeled Consideration of relationship between researcher and participant, Analytic rigor, Involvement of experts by experience, Clear aim to investigate experiences of coercion, and Overall value for this review. Each row provides data for Studies, n (%) and Participants, n (%). Row 1: Consideration of relationship between researcher and participant, Yes, 67 (23), 778 (20.2). Row 2: Consideration of relationship between researcher and participant, Partly, 74 (25.4), 1034 (26.8). Row 3: Consideration of relationship between researcher and participant, No, 150 (51.5), 2041 (53). Row 4: Analytic rigor, High, 46 (15.8), 545 (14.1). Row 5: Analytic rigor, Intermediate, 182 (62.5), 2310 (60). Row 6: Analytic rigor, Low, 63 (21.6), 998 (25.9). Row 7: Involvement of experts by experience, Present in the research team, 41 (15.5), 886 (23). Row 8: Involvement of experts by experience, Consulted in a reference group, 15 (5.7), 147 (3.8). Row 9: Involvement of experts by experience, No/not reported, 209 (78.9), 2820 (73.2). Row 10: Clear aim to investigate experiences of coercion, Clear, 167 (63), 2866 (74.4). Row 11: Clear aim to investigate experiences of coercion, Unclear, 7 (2.6), 75 (1.9). Row 12: Clear aim to investigate experiences of coercion, Other aim than experience of coercion, 91 (34.3), 912 (23.7). Row 13: Overall value for this review, High, 63 (21.6), 957 (24.8). Row 14: Overall value for this review, Intermediate, 132 (45.4), 1771 (46). Row 15: Overall value for this review, Low, 96 (33), 1125 (29.2).

Navigate back to Table 3.

## Table 4 Long description

A table comparing patient experiences of involuntary admission at different times. The table has three columns: Positive, Mixed, and Negative, and four rows including the header. Row 1: Time of data collection relative to involuntary admission (n = 73). Row 2: During involuntary admission (n = 20), Positive: 12 (60), Mixed: 11 (55), Negative: 19 (95). Row 3: After the involuntary admission ended (n = 40), Positive: 25 (62.5), Mixed: 27 (67.5), Negative: 38 (95). Row 4: Mixed (n = 13), Positive: 4 (30.8), Mixed: 4 (30.8), Negative: 11 (84.6).

Navigate back to Table 4.

## Long description

A textbox containing definitions of various coercive practices in mental healthcare. The text defines involuntary admission as admission to hospital sanctioned by law, either against the will of the patient or for those lacking decision-making capacity who represent a risk to themselves or others. Forensic care is described as taking place at the interface between mental healthcare and the penal code, usually decided by the penal system following a serious legal offense by the patient, often involving long-term detention in secure in-patient settings. Coercive measures are defined as measures used to control or reduce the risk of a patient’s aggressive acts or self-harm. Seclusion is explained as separating the patient from other patients and/or staff to various degrees. Restraints are described as restricting movement with mechanical restraints, physical restraints, or chemical restraints. A community treatment order is defined as a legal order mandating a patient to adhere to treatment in the community. Involuntary treatment is described as forced treatment of a patient, usually involving medication aimed at symptom reduction or relapse prevention, but can also include other treatments. Tribunal/review board procedures are defined as formal hearings by an authority or judicial body to safeguard that clinical decisions conform to legal requirements, often following patient appeal or routine re-examination.

Navigate back to .

## Long description

A table categorizing patient experiences with community treatment orders into negative, mixed, and positive experiences. The table has three rows and three columns. The columns are labeled ‘Negative patient experience’, ‘Mixed experience’, and ‘Positive experience’. Row 1: Negative patient experience, others felt strongly that being on a CTO was a negative experience and not about care, but rather about being monitored or under surveillance by the team, which may implicitly feel disempowering: [The CTO] sort of demoralizes you in some aspects because it takes away your choices, your decision-making in some respects. [pause] Its compulsory medication which is not always the right thing I dont believe. 31 Patients explained that they experienced seclusion as being in a prison because in their opinion only prison inmates are locked up and are deprived of freedom of movement. The patients who were imprisoned prior to their experience of being secluded clearly revisited their imprisonment when they were secluded. The memories and negative emotions related to imprisonment were triggered by the similarity for them between a prison cell and a seclusion room. 32. Row 2: Mixed experience, Overall, the sense of lacking choice and control, the emphasis placed on medication and the threat of hospitalisation were more dominant concerns in the consumer interviews but, on balance, having supports was also an important element of being on a CTO for some consumer participants. 33 Being committed against ones will was sometimes experienced as terrible, while at the same time secure and necessary. 34. Row 3: Positive experience, Patients greatly appreciate continuous observation as it seems to achieve the right balance of feeling cared for while still remaining autonomous. Ive had it a few times that a nurse offered me to talk a little or that I could reach out to them for a chat and I liked that very much. In general, that there are people around you and dont harass you and dont want anything but just have a look at what youre doing, how you are. [] Ive always found that pleasant. 35 Although, under s117 of the Mental Health Act, support should be provided according to need, service users tended to equate the CTO with receiving higher levels of support and hence were often keen to stay on it. This was the case for one young man, for example, who described the CTO as a guarantee of support and monitoring (which he felt had been lacking in the past, leading to isolation and deterioration in his mental health): Im on my own. I need someone like a social worker to come and drop off my medication, a doctor to come in every six months to see me, to make sure Im alright. Ive got no-one. People like me need things like that. 36.

Navigate back to .
