# Supplementary material for: Patient experiences of coercion in mental healthcare: systematic review and metasummary of qualitative studies from 1991 to 2025
Source: BJPsych Open. 2026 Aug 6;12(5):e205. doi: 10.1192/bjo.2026.12067 (PMC13458378; doi:10.1192/bjo.2026.12067)
Supplement: Supplementary file 1 [file S2056472426120675sup002.docx]

**Search strategies for
qualitative studies of patient experiences of coercion in psychiatry**

Date searched: 3 June 2025 (initial search 7 October–4 November 2021)

| **Database** | **Number of retrieved records** |
| --- | --- |
| Medline (Ovid) | 13 014 |
| Embase (Ovid) | 17 496 |
| PsycInfo (Ovid) | 12 966 |
| Cinahl (Ebsco) | 7 249 |
| Web of Science (Clarivate) | 11 121 |
| Scpous (Elsevier) | 1 083 |
| Sociological abstracts (ProQuest) | 1 602 |
| Applied Social Sciences Index & Abstracts (ASSIA) (ProQuest) | 3 164 |
| Norart (National Library of Norway) | 408 |
| SveMed+ (Karolinska institute University Library) | 58 |
| Google Scholar | 600 |
| OpenGrey.eu | 24 |

*Three sources; Norart, SveMed+ and OpenGrey.eu, have not been updated since initial search or is not available anymore, and were only searched 2021.*

Database: **MEDLINE ALL (Ovid)** 1946 to May 29, 2025

Date searched: 3 June 2025

Result: 13 014

| **#** | **Searches** | **Results** |
| --- | --- | --- |
| 1 | involuntary commitment/ or "commitment of mentally ill"/ or involuntary treatment/ or involuntary treatment, psychiatric/ or treatment refusal/ or coercion/ or restraint, physical/ or (coerc* or "means of compulsion*" or enforcement measure* or restrain* or constrain* or seclu* or detention* or involuntar* or in-voluntar* or non-voluntar* or nonvoluntar* or ((forced or forcible) adj medic*) or persuasive communicat* or (compuls* adj5 (confine* or detain* or refus* or mandate* or appeal* or advoc* or hospital* or admiss* or admit* or readmiss* or re-admiss* or commit* or releas* or assess* or treat* or intervention* or healthcare or health care or inpatient* or in-patient* or outpatient* or out-patient*)) or ((mandator* or forced or forcible or civil or court-order* or legal*) adj2 (confine* or detain* or refus* or mandate* or appeal* or advoc* or hospital* or admiss* or admit* or readmiss* or re-admiss* or commit* or releas* or assess* or treat* or intervention* or healthcare or health care or inpatient* or in-patient* or outpatient* or out-patient*)) or ((aggressi* or violen*) adj3 (prevent* or control*)) or (closed* adj2 (ward* or unit* or department* or treatment*)) or community treatment order* or assisted outpatient treatment* or assisted out-patient treatment* or outpatient commitment* or out-patient commitment* or conditional release* or mental health act? or mental treatment act? or treatment refusal* or cto or iot or ito or opc or aot or cct).ti,ab,kf. | 406197 |
| 2 | mental health/ or exp mental disorders/ or exp psychiatry/ or exp mental health services/ or exp mentally ill persons/ or (mental* or psychiatr*).ti,ab,kf. | 2103461 |
| 3 | exp qualitative research/ or interviews as topic/ or interview, psychological/ or focus groups/ or narration/ or grounded theory/ or (qualitative or interview* or focus group* or (purpos* adj4 sampl*) or narrat* or (grounded adj (theor* or analys*)) or (observation* adj2 method*) or content analys* or discours* or discurs* or action research or (participa* adj (action or research)) or phenomenolog* or phenomenograph* or ethnograph* or ethnolog* or fieldwork or fieldstud* or (field adj (work or study or studies or research)) or key informant* or account* or semi-structured or semistructured or unstructured or structured or open-ended* or openended* or informal or in-depth or indepth or "face-to-face" or guide or discussion* or theme* or thematic or experience* or experiental or humanistic or existential or ((patient* or personal or client* or user* or stakeholder* or subjective) adj3 (attitude* or perspective* or view* or perception*))).ti,ab,kf. | 4009084 |
| 4 | 1 and 2 and 3 | 15487 |
| 5 | limit 4 to (english or danish or norwegian or swedish) | 14503 |
| 6 | limit 5 to yr="1991 -Current" | 13944 |
| 7 | 6 not (exp child/ not exp adult/) | 13014 |

Database: **Embase (Ovid)** 1974 to 2025 May 30

Date searched: 3 June 2025

Result: 17 496

| **#** | **Searches** | **Results** |
| --- | --- | --- |
| 1 | involuntary commitment/ or treatment refusal/ or coercion/ or physical restraint/ or detention/ or persuasive communication/ or coerc*.dq. or (coerc* or "means of compulsion*" or enforcement measure* or restrain* or constrain* or seclu* or detention* or involuntar* or in-voluntar* or non-voluntar* or nonvoluntar* or ((forced or forcible) adj medic*) or persuasive communicat* or (compuls* adj5 (confine* or detain* or refusal* or mandate* or appeal* or advoc* or hospital* or admiss* or admit* or readmiss* or re-admiss* or commit* or releas* or assess* or treat* or intervention* or healthcare or health care or inpatient* or in-patient* or outpatient* or out-patient*)) or ((mandator* or forced or forcible or civil or court-order* or legal*) adj2 (confine* or detain* or refusal* or mandate* or appeal* or advoc* or hospital* or admiss* or admit* or readmiss* or re-admiss* or commit* or releas* or assess* or treat* or intervention* or healthcare or health care or inpatient* or in-patient* or outpatient* or out-patient*)) or ((aggressi* or violen*) adj3 (prevent* or control*)) or (closed* adj2 (ward* or unit* or department* or treatment*)) or community treatment order* or assisted outpatient treatment* or assisted out-patient treatment* or outpatient commitment* or out-patient commitment* or conditional release* or mental health act? or mental treatment act? or treatment refusal* or cto or iot or ito or opc or aot or cct).ti,ab,kf. | 481514 |
| 2 | exp mental health/ or exp mental disease/ or exp psychiatry/ or mental patient/ or exp mental health care/ or (mental* or psychiatr*).ti,ab,kf. | 3560618 |
| 3 | exp qualitative research/ or exp interview/ or psychological interview/ or narrative/ or grounded theory/ or exp observational method/ or content analysis/ or discourse analysis/ or thematic analysis/ or exp action research/ or phenomenology/ or ethnography/ or ethnology/ or field work/ or field study/ or open ended questionnaire/ or experience/ or patient attitude/ or (qualitative or interview* or focus group* or (purpos* adj4 sampl*) or narrat* or (grounded adj (theor* or analys*)) or (observation* adj2 method*) or content analys* or discours* or discurs* or action research or (participa* adj (action or research)) or phenomenolog* or phenomenograph* or ethnograph* or ethnolog* or fieldwork or fieldstud* or (field adj (work or study or studies or research)) or key informant* or account* or semi-structured or semistructured or unstructured or structured or open-ended* or openended* or informal or in-depth or indepth or "face-to-face" or guide or discussion* or theme* or thematic or experience* or experiental or humanistic or existential or ((patient* or personal or client* or user* or stakeholder* or subjective) adj3 (attitude* or perspective* or view* or perception*))).ti,ab,kf. | 5654019 |
| 4 | 1 and 2 and 3 | 26264 |
| 5 | limit 4 to (english or danish or norwegian or swedish) | 24981 |
| 6 | limit 5 to yr="1991 -Current" | 24410 |
| 7 | 6 not (exp child/ not exp adult/) | 22435 |
| 8 | limit 7 to conference abstracts | 4939 |
| 9 | 7 not 8 | 17496 |

Database: **APA PsycInfo (Ovid)** 1806 to May 2025 Week 4

Date searched: 3 June 2025

Result: 12 966

| **#** | **Searches** | **Results** |
| --- | --- | --- |
| 1 | "commitment (psychiatric)"/ or outpatient commitment/ or involuntary treatment/ or treatment refusal/ or coercion/ or physical restraint/ or persuasive communication/ or patient seclusion/ or (coerc* or "means of compulsion*" or enforcement measure* or restrain* or constrain* or seclu* or detention* or involuntar* or in-voluntar* or non-voluntar* or nonvoluntar* or ((forced or forcible) adj medic*) or persuasive communicat* or (compuls* adj5 (confine* or detain* or refusal* or mandate* or appeal* or advoc* or hospital* or admiss* or admit* or readmiss* or re-admiss* or commit* or releas* or assess* or treat* or intervention* or healthcare or health care or inpatient* or in-patient* or outpatient* or out-patient*)) or ((mandator* or forced or forcible or civil or court-order* or legal*) adj2 (confine* or detain* or refusal* or mandate* or appeal* or advoc* or hospital* or admiss* or admit* or readmiss* or re-admiss* or commit* or releas* or assess* or treat* or intervention* or healthcare or health care or inpatient* or in-patient* or outpatient* or out-patient*)) or ((aggressi* or violen*) adj3 (prevent* or control*)) or (closed* adj2 (ward* or unit* or department* or treatment*)) or community treatment order* or assisted outpatient treatment* or assisted out-patient treatment* or outpatient commitment* or out-patient commitment* or conditional release* or mental health act? or mental treatment act? or treatment refusal* or cto or iot or ito or opc or aot or cct).ti,ab,id. | 150527 |
| 2 | exp mental health/ or exp mental disorders/ or exp psychiatry/ or psychiatric patients/ or exp mental health services/ or (mental* or psychiatr*).ti,ab,id. | 1578657 |
| 3 | qualitative methods/ or exp focus group/ or grounded theory/ or interpretative phenomenological analysis/ or narrative analysis/ or semi-structured interview/ or thematic analysis/ or exp interviews/ or exp observation methods/ or exp content analysis/ or action research/ or phenomenology/ or ethnography/ or ethnology/ or client attitudes/ or (qualitative or interview* or focus group* or (purpos* adj4 sampl*) or narrat* or (grounded adj (theor* or analys*)) or (observation* adj2 method*) or content analys* or discours* or discurs* or action research or (participa* adj (action or research)) or phenomenolog* or phenomenograph* or ethnograph* or ethnolog* or fieldwork or fieldstud* or (field adj (work or study or studies or research)) or key informant* or account* or semi-structured or semistructured or unstructured or structured or open-ended* or openended* or informal or in-depth or indepth or "face-to-face" or guide or discussion* or theme* or thematic or experience* or experiental or humanistic or existential or ((patient* or personal or client* or user* or stakeholder* or subjective) adj3 (attitude* or perspective* or view* or perception*))).ti,ab,id. | 1957531 |
| 4 | 1 and 2 and 3 | 16358 |
| 5 | limit 4 to (english or danish or norwegian or swedish) | 15041 |
| 6 | limit 5 to yr="1991 -Current" | 14027 |
| 7 | 6 not (("100" or "200") not "300").ag. | 12966 |

Database: **Cinahl (EBSCOhost)**

Date searched: 3 June 2025

Result: 7 249

| **#** | **Searches** | **Results** |
| --- | --- | --- |
| S1 | (MH "Involuntary Commitment") or (MH "involuntary treatment") or (MH "treatment refusal") or (MH coercion) or (MH "restraint, physical") or (MH "persuasive communication") or (MH "patient seclusion") OR (XB ( coerc* or "means of compulsion*" or enforcement measure* or restrain* or constrain* or seclu* or detention* or involuntar* or in-voluntar* or non-voluntar* or nonvoluntar* or ((forced or forcible) W0 medic*) or persuasive communicat* or (compuls* N4 (confine* or detain* or refusal* or mandate* or appeal* or advoc* or hospital* or admiss* or admit* or readmiss* or re-admiss* or commit* or releas* or assess* or treat* or intervention* or healthcare or health care or inpatient* or in-patient* or outpatient* or out-patient*)) or ((mandator* or forced or forcible or civil or court-order* or legal*) N1 (confine* or detain* or refusal* or mandate* or appeal* or advoc* or hospital* or admiss* or admit* or readmiss* or re-admiss* or commit* or releas* or assess* or treat* or intervention* or healthcare or health care or inpatient* or in-patient* or outpatient* or out-patient*)) or ((aggressi* or violen*) N2 (prevent* or control*)) or (closed* N1 (ward* or unit* or department* or treatment*)) or community treatment order* or assisted outpatient treatment* or assisted out-patient treatment* or outpatient commitment* or out-patient commitment* or conditional release* or mental health act? or mental treatment act? or treatment refusal* or cto or iot or ito or opc or aot or cct ) | 77,961 |
| S2 | (MH "mental health") or (MH "mental disorders+" or (MH "Psychiatry") or (MH "psychiatric patients") or (MH "mental health services+") OR (TI (mental* or psychiatr*) or AB (mental* or psychiatry*)) | 910,084 |
| S3 | (MH "qualitative studies+") or (MH "interviews+") or (MH "narratives") or (MH "observational methods+") or (MH "content analysis") or (MH "discourse analysis") or (MH "thematic analysis") or (MH "phenomenology") or (MH "Phenomenological Research") or (MH "Ethnology") or ("MH field studies") or (MH "Open-Ended Questionnaires") or (MH "focus groups") or (MH "purposive sample") or (MH "patient attitudes") OR (XB ( qualitative or interview* or focus group* or (purpos* N3 sampl*) or narrat* or (grounded W0 (theor* or analys*)) or (observation* N1 method*) or content analys* or discours* or discurs* or action research or (participa* W0 (action or research)) or phenomenolog* or phenomenograph* or ethnograph* or ethnolog* or fieldwork or fieldstud* or (field W0 (work or study or studies or research)) or key informant* or account* or semi-structured or semistructured or unstructured or structured or open-ended* or openended* or informal or in-depth or indepth or "face-to-face" or guide or discussion* or (theme* or thematic) or experience* or experiental or humanistic or existential or ((patient* or personal or client* or user* or stakeholder* or subjective) N2 (attitude* or perspective* or view* or perception*)) ) ) | 1,412,955 |
| S4 | S1 and S2 and S3 | 7,995 |
| S5 | S1 and S2 and S3  *Limiters:*   - *Language: Danish, English, Norwegian, Swedish* - *Published Date: 19910101-20251231* | 7,776 |
| S6 | S5 NOT ((MH "Child+") NOT (MH "Adult+")) | 7,249 |

Database: **Web of Science Core Collection (Clarivate)**

- Science Citation Index Expanded (SCI-EXPANDED) 1945 to present

- Science Citation Index Expanded (SSCI) 1956 to present
- Arts & Humanities Citation Index (AHCI) 1975 to present

- Emerging Sources Citation Index (ESCI) 2020 to present

Date searched: 3 June 2025

Result: 11 121

| **#** | **Searches** | **Results** |
| --- | --- | --- |
| #1 | TS=(coerc* or "means of compulsion*" or "enforcement measure*" or restrain* or constrain* or seclu* or detention* or involuntar* or "in-voluntar*" or "non-voluntar*" or nonvoluntar* or ((forced or forcible) NEAR/0 medic*) or "persuasive communicat*" or (compuls* NEAR/4 (confine* or detain* or refusal* or mandate* or appeal* or advoc* or hospital* or admiss* or admit* or readmiss* or "re-admiss*" or commit* or releas* or assess* or treat* or intervention* or healthcare or "health care" or inpatient* or "in-patient*" or outpatient* or "out-patient*")) or ((mandator* or forced or forcible or civil or "court-order*" or legal*) NEAR/1 (confine* or detain* or refusal* or mandate* or appeal* or advoc* or hospital* or admiss* or admit* or readmiss* or "re-admiss*" or commit* or releas* or assess* or treat* or intervention* or healthcare or "health care" or inpatient* or "in-patient*" or outpatient* or "out-patient*")) or ((aggressi* or violen*) NEAR/2 (prevent* or control*)) or (closed* NEAR/1 (ward* or unit* or department* or treatment*)) or "community treatment order*" or "assisted outpatient treatment*" or "assisted out-patient treatment*" or "outpatient commitment*" or "out-patient commitment*" or "conditional release*" or "mental health act?" or "mental treatment act?" or "treatment refusal*" or cto or iot or ito or opc or aot or cct) | 1,438,116 |
| #2 | TS=(mental* or psychiatr*) | 1,025,272 |
| #3 | TS=(qualitative or interview* or focus group* or (purpos* NEAR/3 sampl*) or narrat* or (grounded NEAR/0 (theor* or analys*)) or (observation* NEAR/1 method*) or content analys* or discours* or discurs* or action research or (participa* NEAR/0 (action or research)) or phenomenolog* or phenomenograph* or ethnograph* or ethnolog* or fieldwork or fieldstud* or (field NEAR/0 (work or study or studies or research)) or key informant* or account* or semi-structured or semistructured or unstructured or structured or open-ended* or openended* or informal or in-depth or indepth or "face-to-face" or guide or discussion* or theme* or thematic or experience* or experiental or humanistic or existential or ((patient* or personal or client* or user* or stakeholder* or subjective) NEAR/2 (attitude* or perspective* or view* or perception*))) | 14,551,847 |
| #4 | #1 AND #2 AND #3 | 13,771 |
| #5 | #4 AND LA=("ENGLISH" OR "DANISH" OR "NORWEGIAN") | 12,989 |
| #6 | #5 AND PY=1991-2025 | 12,876 |
| #7 | #6 NOT TS=(child* NOT adult*) | 11,121 |

Database: **Scopus (Elsevier)**

Date searched: 3 June 2025

Result: 1 083

| **#** | **Searches** | **Results** |
| --- | --- | --- |
| 1 | TITLE-ABS-KEY ( coerc* OR "means of compulsion*" OR "enforcement measure*" OR restrain* OR constrain* OR seclu* OR detention* OR involuntar* OR "in-voluntar*" OR "non-voluntar*" OR nonvoluntar* OR ( ( forced OR forcible ) W/0 medic* ) OR "persuasive communicat*" OR ( compuls* W/4 ( confine* OR detain* OR refusal* OR mandate* OR appeal* OR advoc* OR hospital* OR admiss* OR admit* OR readmiss* OR "re-admiss*" OR commit* OR releas* OR assess* OR treat* OR intervention* OR healthcare OR "health care" OR inpatient* OR "in-patient*" OR outpatient* OR "out-patient*" ) ) OR ( ( mandator* OR forced OR forcible OR civil OR "court-order*" OR legal* ) W/1 ( confine* OR detain* OR refusal* OR mandate* OR appeal* OR advoc* OR hospital* OR admiss* OR admit* OR readmiss* OR "re-admiss*" OR commit* OR releas* OR assess* OR treat* OR intervention* OR healthcare OR "health care" OR inpatient* OR "in-patient*" OR outpatient* OR "out-patient*" ) ) OR ( ( aggressi* OR violen* ) W/2 ( prevent* OR control* ) ) OR ( closed* W/1 ( ward* OR unit* OR department* OR treatment* ) ) OR "community treatment order*" OR "assisted outpatient treatment*" OR "assisted out-patient treatment*" OR "outpatient commitment*" OR "out-patient commitment*" OR "conditional release*" OR "mental health act?" OR "mental treatment act?" OR "treatment refusal*" OR cto OR iot OR ito OR opc OR aot OR cct ) | 2,445,275 |
| 2 | TITLE-ABS ( mental* OR psychiatr* ) OR AUTHKEY ( mental* OR psychiatr* ) | 1,808,366 |
| 3 | TITLE-ABS-KEY ( qualitative OR interview* OR focus AND group* OR ( purpos* W/3 sampl* ) OR narrat* OR ( grounded W/0 ( theor* OR analys* ) ) OR ( observation* W/1 method* ) OR content AND analys* OR discours* OR discurs* OR action AND research OR ( participa* W/0 ( action OR research ) ) OR phenomenolog* OR phenomenograph* OR ethnograph* OR ethnolog* OR fieldwork OR fieldstud* OR ( field PRE/0 ( work OR study OR studies OR research ) ) OR key AND informant* OR account* OR semi-structured OR semistructured OR unstructured OR structured OR open-ended* OR openended* OR informal OR in-depth OR indepth OR "face-to-face" OR guide OR discussion* OR ( theme* OR thematic ) OR experience* OR experiental OR humanistic OR existential OR ( ( patient* OR personal OR client* OR user* OR stakeholder* OR subjective ) W/2 ( attitude* OR perspective* OR view* OR perception* ) ) ) | 211,650 |
| 4 | 1 AND 2 AND 3 | 1,121 |
| 5 | 4 AND PUBYEAR > 1990 AND PUBYEAR < 2026 AND ( LIMIT-TO ( LANGUAGE , "English" ) ) | 1,083 |

Database: **Sociological Abstracts (ProQuest)**

Date searched: 3 June 2025

Result: 1 602

| **#** | **Searches** | **Results** |
| --- | --- | --- |
| S1 | MAINSUBJECT.EXACT("Coercion") OR TI,AB,IF(coerc* OR "means of compulsion*" OR "enforcement measure*" OR restrain* OR constrain* OR seclu* OR detention* OR involuntar* OR "in-voluntar*" OR "non-voluntar*" OR nonvoluntar* OR ((forced OR forcible) PRE/0 medic*) OR "persuasive communicat*" OR (compuls* NEAR/4 (confine* OR detain* OR refusal* OR mandate* OR hospital* OR admission OR admissions OR admit OR admitted OR readmission OR readmissions OR "re-admission" OR "re-admissions" OR commit OR comitted OR releas* OR assess* OR treat* OR intervention* OR healthcare OR "health care" OR inpatient* OR "in-patient" OR "in-patients" OR outpatient* OR "out-patient" OR "out-patients")) OR ((mandator* OR forced OR forcible OR civil OR court-order* OR legal*) NEAR/1 (confine* OR detain* OR refusal* OR mandate* OR hospital* OR admiss* OR admit* OR readmiss* OR re-admiss*)) OR ((mandator* OR forced OR forcible OR civil OR court-order* OR legal*) NEAR/1 (commit* OR releas* OR assess* OR treat* OR intervention* OR healthcare OR "health care" OR inpatient* OR in-patient* OR outpatient* OR out-patient*)) OR ((aggressi* OR violen*) NEAR/2 (prevent* OR control*)) OR (closed* NEAR/1 (ward* OR unit* OR department* OR treatment*)) OR "community treatment order*" OR "assisted outpatient treatment*" OR "assisted out-patient treatment*" OR "outpatient commitment" OR "out-patient commitment*" OR "conditional release" OR "mental health act?" OR "mental treatment act?" OR "treatment refusal" OR "treatment refusals" OR cto OR iot OR ito OR opc OR aot OR cct) | 56,996 |
| S2 | MAINSUBJECT.EXACT("Mental Health") OR MAINSUBJECT.EXACT.EXPLODE("Mental disorders") OR MAINSUBJECT.EXACT.EXPLODE("Psychiatry") OR MAINSUBJECT.EXACT("Mental Patients") OR MAINSUBJECT.EXACT("Mental Health Services") OR TI,AB,IF(mental* OR psychiatr*) | 87,633 |
| S3 | MAINSUBJECT.EXACT("Qualitative Methods") OR MAINSUBJECT.EXACT("Interviews") OR MAINSUBJECT.EXACT("Narratives") OR MAINSUBJECT.EXACT("Grounded Theory") OR MAINSUBJECT.EXACT("Content Analysis") OR MAINSUBJECT.EXACT("Discourse Analysis") OR MAINSUBJECT.EXACT("Action Research") OR MAINSUBJECT.EXACT("Phenomenology") OR MAINSUBJECT.EXACT("Ethnology") OR MAINSUBJECT.EXACT("Ethnography") OR MAINSUBJECT.EXACT("Fieldwork") OR MAINSUBJECT.EXACT("Experience") OR TI,AB,IF(qualitative OR interview* OR focus group* OR (purpos* NEAR/3 sampl*) OR narrat* OR (grounded NEAR/0 (theor* OR analys*)) OR (observation* NEAR/1 method*) OR content analys* OR discours* OR discurs* OR action research OR (participa* PRE/0 (action OR research)) OR phenomenolog* OR phenomenograph* OR ethnograph* OR ethnolog* OR fieldwork OR fieldstud* OR (field PRE/0 (work OR study OR studies OR research)) OR key informant* OR account* OR semi-structured OR semistructured OR unstructured OR structured OR open-ended* OR openended* OR informal OR in-depth OR indepth OR "face-to-face" OR guide OR discussion* OR (theme* OR thematic) OR experience* OR experiental OR humanistic OR existential OR ((patient* OR personal OR client* OR user* OR stakeholder* OR subjective) NEAR/2 (attitude* OR perspective* OR view* OR perception*))) | 806,629 |
| S4 | [S1] OR [S2] OR [S3] | 1,912 |
| S5 | ([S1] AND [S2] AND [S3]) AND (la.exact("ENG" OR "NOR" OR "SWE") AND pd(19910101-20251231) | 1,602 |

Database: **ASSIA (ProQuest)**

Date searched: 3 June 2025

Result: 3 164

| **#** | **Searches** | **Results** |
| --- | --- | --- |
| S1 | MAINSUBJECT.EXACT("Coercion") OR TI,AB,IF(coerc* OR "means of compulsion*" OR "enforcement measure*" OR restrain* OR constrain* OR seclu* OR detention* OR involuntar* OR "in-voluntar*" OR "non-voluntar*" OR nonvoluntar* OR ((forced OR forcible) PRE/0 medic*) OR "persuasive communicat*" OR (compuls* NEAR/4 (confine* OR detain* OR refusal* OR mandate* OR hospital* OR admission OR admissions OR admit OR admitted OR readmission OR readmissions OR "re-admission" OR "re-admissions" OR commit OR comitted OR releas* OR assess* OR treat* OR intervention* OR healthcare OR "health care" OR inpatient* OR "in-patient" OR "in-patients" OR outpatient* OR "out-patient" OR "out-patients")) OR ((mandator* OR forced OR forcible OR civil OR court-order* OR legal*) NEAR/1 (confine* OR detain* OR refusal* OR mandate* OR hospital* OR admiss* OR admit* OR readmiss* OR re-admiss*)) OR ((mandator* OR forced OR forcible OR civil OR court-order* OR legal*) NEAR/1 (commit* OR releas* OR assess* OR treat* OR intervention* OR healthcare OR "health care" OR inpatient* OR in-patient* OR outpatient* OR out-patient*)) OR ((aggressi* OR violen*) NEAR/2 (prevent* OR control*)) OR (closed* NEAR/1 (ward* OR unit* OR department* OR treatment*)) OR "community treatment order*" OR "assisted outpatient treatment*" OR "assisted out-patient treatment*" OR "outpatient commitment" OR "out-patient commitment*" OR "conditional release" OR "mental health act?" OR "mental treatment act?" OR "treatment refusal" OR “treatment refusals” OR cto OR iot OR ito OR opc OR aot OR cct) | 32,740 |
| S2 | MAINSUBJECT.EXACT("Mental Health") OR MAINSUBJECT.EXACT.EXPLODE("Mental disorders") OR MAINSUBJECT.EXACT.EXPLODE("Psychiatry") OR MAINSUBJECT.EXACT.EXPLODE("Mental health services") OR TI,AB,IF(mental* or psychiatr*) | 220,154 |
| S3 | MAINSUBJECT.EXACT("Qualitative methods") OR MAINSUBJECT.EXACT("Qualitative research") OR MAINSUBJECT.EXACT("Interviews") OR MAINSUBJECT.EXACT("Focus group interviews") OR MAINSUBJECT.EXACT("Semistructured interviews") OR MAINSUBJECT.EXACT("Semistructured questionnaires") OR MAINSUBJECT.EXACT.EXPLODE("Narratives") OR MAINSUBJECT.EXACT("Content analysis") OR MAINSUBJECT.EXACT.EXPLODE("Action research") OR MAINSUBJECT.EXACT("Ethnography") OR MAINSUBJECT.EXACT("Grounded theory") OR MAINSUBJECT.EXACT("Discourse analysis") OR MAINSUBJECT.EXACT.EXPLODE("Phenomenology") OR MAINSUBJECT.EXACT("Fieldwork") OR MAINSUBJECT.EXACT("Focus groups") OR MAINSUBJECT.EXACT.EXPLODE("Experiences") OR TI,AB,IF(qualitative OR interview* OR focus group* OR (purpos* NEAR/3 sampl*) OR narrat* OR (grounded NEAR/0 (theor* OR analys*)) OR (observation* NEAR/1 method*) OR content analys* OR discours* OR discurs* OR action research OR (participa* PRE/0 (action OR research)) OR phenomenolog* OR phenomenograph* OR ethnograph* OR ethnolog* OR fieldwork OR fieldstud* OR (field PRE/0 (work OR study OR studies OR research)) OR key informant* OR account* OR semi-structured OR semistructured OR unstructured OR structured OR open-ended* OR openended* OR informal OR in-depth OR indepth OR "face-to-face" OR guide OR discussion* OR (theme* OR thematic) OR experience* OR experiental OR humanistic OR existential OR ((patient* OR personal OR client* OR user* OR stakeholder* OR subjective) NEAR/2 (attitude* OR perspective* OR view* OR perception*))) | 543,306 |
| S4 | [S1] AND [S2] AND [S3] | 3,197 |
| S5 | ([S1] AND [S2] AND [S3]) AND (la.exact("ENG") AND pd(19910101-20251231)) | 3,164 |

Database: **Norart (Norwegian and Nordic index to periodical articles) (National Library of Norway)**

Date searched: 31 October 2021 *(database not updated after 2020)*

Result: 408

| **Search field** | **Word Search** |
| --- | --- |
| Search string | (tvang* or tvung* or restriksjon* or skjerming* or forvaring* or samtykke*)  and (mental* or psyk*) |
| Results | 408 |

Database: **SveMed+ (Karolinska Institutet University Library)**

Date searched: 31 October 2021 *(database not updated after 2019)*

Result: 58

| **#** | **Searches** | **Results** |
| --- | --- | --- |
| 1 | noexp:"Commitment of Mentally Ill" | 419 |
| 2 | noexp:"Involuntary Treatment" | 3 |
| 3 | noexp:"Involuntary Treatment, Psychiatric" | 36 |
| 4 | noexp:"Treatment Refusal" | 110 |
| 5 | exp:"Coercion" | 248 |
| 6 | noexp:"Restraint, Physical" | 134 |
| 7 | coerc* OR involuntar* OR "in-voluntar*" OR restrain* OR compuls* OR mandator* OR forced OR forcible OR tving* OR tvung* OR tvång* | 1488 |
| 8 | #1 OR #2 OR #3 OR #4 OR #5 OR #6 OR #7 | 1585 |
| 9 | noexp:"mental health" | 610 |
| 10 | exp:"Mental Disorders" | 13898 |
| 11 | exp:"Psychiatry" | 2951 |
| 12 | exp:"Mental Health Services" | 2193 |
| 13 | exp:"Mentally Ill Persons" | 79 |
| 14 | mental* OR psychiatr* OR psykisk* | 9290 |
| 15 | #9 OR #10 OR #11 OR #12 OR #13 OR #14 | 18472 |
| 16 | exp:"Qualitative Research" | 1464 |
| 17 | noexp:"Interviews as Topic" | 813 |
| 18 | noexp:"Interview, Psychological" | 165 |
| 19 | noexp:"Focus Groups" | 946 |
| 20 | noexp:"Narration" | 857 |
| 21 | noexp:"Grounded Theory" | 30 |
| 22 | qualitative* OR interview* OR "focus group*" OR narrat* OR "grounded theory" OR "observation method" OR "content analysis" OR discours* OR discurs* OR "action research" OR phenomeno* OR ethnograph* OR ethnolog* OR "open-ended" OR unstructured OR "un-structured" OR "semi-structured" OR semistructured OR fritext OR fritekst OR kvalitativ* | 6168 |
| 23 | #16 OR #17 OR #18 OR #19 OR #20 OR #21 OR #22 | 6176 |
| 24 | #8 AND #15 AND #23 | 58 |

**Google Scholar**

Date searched: 3 June 2025

Result: 600 (of about 1 610 000 results)

| Search string | coercion\|coercive\|coerced\|coercing\|compulsion\|involuntary\|involuntariness\|"treatment refusal" mental\|mentally\|psychiatry\|psychiatric qualitative\|experience\|narrative\|perspective\|perception |
| --- | --- |
| Results | 600 |
| Settings/  selection | Results sorted by relevance.  4 Nov 2021: First 500 of about 995 000 results.  3 Jun 2025: First 100 published between 2021 and 2025, of about 1 610 000 results. |

# Grey literature

**OpenGrey.eu**

Date searched: 04.11.2021 *(database closed in 2021 and is no longer available)*

Result: 24

| Search string | (coerc* OR involuntar* OR restrain* OR compuls* OR mandator* OR forced OR forcible) AND (mental* OR psychiatry*) AND (qualitative* OR interview* OR "focus group*" OR narrat* OR "grounded theory" OR "observation method" OR "content analysis" OR discours* OR discurs* OR "action research" OR phenomeno* OR ethnograph* OR ethnolog* OR "open-ended" OR unstructured OR "semi-structured") |
| --- | --- |
| Results | 24 hits |
